# Early Detection of Subclinical Myocardial Dysfunction in Familial Dilated Cardiomyopathy Using Myocardial Work Analysis

**DOI:** 10.3390/diagnostics15182363

**Published:** 2025-09-17

**Authors:** Apostolos Vrettos, Ricardo Prista Monteiro, Miltiadis Triantafyllou, Uzma Gul, Sanjeev Bhattacharyya, Luís R. Lopes, Alexios Antonopoulos, Alexandros Protonotarios, Guy Lloyd, Thomas Gossios, Konstantinos Savvatis

**Affiliations:** 1 Barts Heart Centre, St Bartholomew’s Hospital, Barts Health NHS Trust, London EC1A 7BE, UK; 2 Clinical Cardiology at Halmstad Hospital, SE-301 85 Halmstad, Sweden; 3 William Harvey Research Institute, John Vane Science Centre, Queen Mary University of London, Charterhouse Square, London EC1M 6BQ, UK; 4 Institute of Cardiovascular Science, University College London, London WC1E 6BT, UK; 5 NIHR University College London Hospitals Biomedical Research Centre, London NW1 2PB, UK; 6 Inherited Cardiovascular Diseases Unit, St Bartholomew’s Hospital, London EC1A 7BE, UK; 7 Centre for Heart Muscle Disease, Institute of Cardiovascular Science, University College London, London WC1E 6BT, UK; 8 First Cardiology Department, National and Kapodistrian University of Athens, 10679 Athens, Greece; 9 First Cardiology Department, University General Hospital of Thessaloniki AHEPA, 54636 Thessaloniki, Greece

**Keywords:** familial dilated cardiomyopathy, myocardial work, subclinical cardiomyopathy, echocardiography, genetic testing

## Abstract

**Background/Objectives**: Early detection of familial dilated cardiomyopathy (DCM) is crucial for initiating timely interventions. Myocardial work (MW) analysis, which integrates strain imaging and blood pressure, shows promise in identifying subclinical disease. To assess the utility of MW in detecting early myocardial changes in relatives of DCM patients with a positive genotype (G+) compared to genotype-negative (G−) controls. **Methods**: This study involved asymptomatic relatives of DCM patients who underwent comprehensive echocardiographic evaluation, including MW analysis. Subjects (N = 77) were classified into G+ (*n* = 39) and (*n* = 38) groups based on genetic testing. Myocardial work parameters—myocardial global work index (GWI), global constructive work (GCW), global wasted work (GWW), and global work efficiency (GWE)—were measured. Statistical analyses compared these parameters between groups and assessed their predictive value for genotype status. Follow-up data were collected and analysed accordingly. **Results**: Among 77 participants (mean age 36 ± 14 years; 49% women), there were no significant differences in baseline characteristics between G+ and G− groups. S’ septal, s′ average, e′ lateral, E max and E/A were found to be significantly different between the two groups. G+ individuals had significantly reduced GWE (94% vs. 96%, *p* < 0.001) and increased GWW (113 mmHg% vs. 80 mmHg%, *p* = 0.001). After adjustment for significant echocardiographic parameters, GWE (OR = 0.746, 95% CI: 0.560–0.994, *p* = 0.045) and GWW (OR = 1.012, 95% CI: 1.002–1.024, *p* = 0.047) remained significant predictors of gene carrier status in multivariable analysis. The addition of GWE and GWW significantly increased the area under the curve of a model identifying G+ individuals (*p* < 0.05). During a median period of follow-up of 53 months, 16 (21%) individuals expressed a cardiomyopathy phenotype. There was a significant correlation between increased baseline GWW, reduced GWE, and the expression of cardiomyopathy phenotype. **Conclusions**: Myocardial work analysis, specifically GWE and GWW, identifies early myocardial dysfunction in asymptomatic carriers of genetic variants for DCM. These findings suggest that MW could complement traditional imaging in the early detection and management of familial DCM.

## 1. Introduction

Dilated cardiomyopathy (DCM) is a primary myocardial disease characterized by left ventricular dilation and systolic dysfunction, frequently leading to heart failure and arrhythmias. Familial DCM accounts for a significant proportion of cases and is often caused by pathogenic genetic variants affecting structural or functional components of the myocardium. Early detection of genetically predisposed individuals, such as affected family relatives of patients with dilated cardiomyopathy (DCM), is essential in order to guide a more targeted follow-up and initiate early treatment [1]. Myocardial work analysis is a novel method which integrates strain imaging and blood pressure and has the potential to assess myocardial performance. This method quantifies the energy expenditure of the myocardium during contraction and relaxation, providing insights into mechanical function that conventional assessments may overlook. Given that strain imaging alone has shown utility in detecting early myocardial dysfunction, the addition of myocardial work analysis may enhance sensitivity in identifying DCM at an earlier stage. This study aims to determine whether myocardial work analysis can identify subclinical myocardial dysfunction in at-risk relatives of DCM patients, thereby enabling earlier intervention and improved outcomes.

## 2. Materials and Methods

### 2.1. Study Population

Data from consecutive first-degree relatives of DCM patients attending for clinical and echocardiographic evaluation at our institution (Barts Heart Centre, St Bartholomew’s Hospital, Barts Health NHS Trust) were collected. Initially, pedigree analysis and sequencing of a 120 cardiomyopathy-associated gene panel (Appendix A) were performed in all index patients to determine genetic status. Genetic counselling, testing, and analysis were performed as described previously [1]. Variant classification followed the American College of Medical Genetics and Genomics (ACMG) and Association for Molecular Pathology (AMP) guidelines [2]. Likely pathogenic/pathogenic (LP/P) DCM variants were defined as confirmed class 4 or 5 variants. Participants’ data were entered in a prospective database, including demographic, clinical, and echocardiographic parameters.

Inclusion criteria were as follows:

1. Asymptomatic status with either positive (G+) or negative (G−) genotype (no history of heart failure symptoms, and no activity limitation as per clinical records).
2. No echocardiographic evidence of left ventricular dilatation, defined as left ventricular (LV) end-diastolic dimension (EDD) of >56 mm and/or LV end-diastolic volume indexed (EDVi) > 79 mL/m^2^ in males, and LV EDD > 51 mm and/or LV EDVi > 70 mL/m^2^ in females, or systolic impairment with a LV ejection fraction (EF) measured on echocardiography using Simpson’s biplane of <53%.
3. Available genetic screening results.

Patients with prosthetic valve(s); paced rhythm; abnormal LV dimensions or systolic function on echocardiography or cardiac MRI (CMR); more than mild valvular heart disease, uncontrolled cardiovascular risk factors; or those with evidence of target-organ damage, coronary artery disease, and poor echocardiographic image quality were excluded. Individuals with well-controlled hypertension or diabetes on stable therapy (≥3 months) were eligible, reflecting the real-world prevalence of these conditions in relatives. This study was performed according to the Declaration of Helsinki. All assessed patients provided written informed consent to provide anonymized clinical data, as per standard protocol approved by the local Research Ethics Committee (London—Surrey Research Ethics Committee). Approval Code: 227168. Approval Date: 22 March 2018.

### 2.2. Transthoracic Echocardiography

A comprehensive transthoracic 2D echocardiographic study was performed for all subjects using GE Vivid E95 machines in our Echocardiography lab by a cardiac sonography expert blinded to the genetic status of the patients.

Electrocardiographically triggered echocardiographic data were acquired and digitally stored in cine-loop format for offline analysis. Parasternal, apical, and subcostal views were used to acquire two-dimensional, colour, pulsed-wave, and continuous-wave Doppler data. Traditional echocardiographic parameters were measured following the European Association of Cardiovascular Imaging (EACVI) guidelines [3,4]. Assessment of LV mitral valve inflow and tissue annular velocities was conducted according to the relevant guidelines.

Global LV myocardial work was measured as previously described [5]. Left ventricular longitudinal deformation was assessed by measuring global longitudinal strain (GLS) as the average longitudinal strain across 18 segments obtained from apical 4-, 2-, and 3-chamber views at a frame rate of ≥60 frames per second using dedicated software (EchoPac PC, GE Healthcare, Pollards Wood, UK). The timing of aortic and mitral valve opening and closure was manually defined on 2D parasternal or apical long-axis views to simultaneously visualize the mitral and aortic valves in order to define the isovolumetric contraction (IVC) and relaxation (IVR).

**Figure 1:**
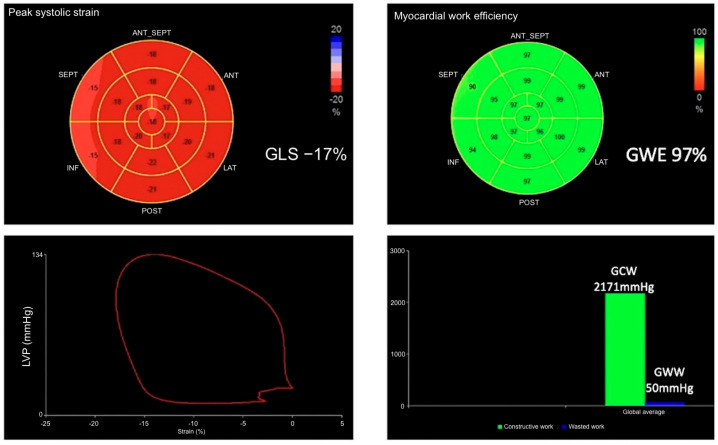
Measurement of myocardial work index derived from LV pressure and strain via echocardiography. From left to right and top to bottom: bullseye diagram of GLS calculated at −17%; bullseye diagram of GWE at a blood pressure of 134/79 mmHg; LV pressure-strain loop; bar graph showing GCW and GWW and the resultant myocardial work indexes.

Brachial blood pressure (BP) was measured in triplicate using a validated automated oscillometric device (appropriate cuff size), after ≥5 min of seated rest, with the arm supported at heart level and no caffeine, exercise, or smoking for ≥30 min. The mean of the last two readings was used for the myocardial work study. Measurements were performed contemporaneously with image acquisition. A dedicated echocardiographic software provided pressure–strain loops by synchronizing longitudinal strain, blood pressure, and data regarding the time of valvular events (Figure 1). Parameters of myocardial work measured included the global work index (GWI), which is the total work within the area of the LV pressure–strain loop calculated from mitral valve closure to mitral valve opening; global constructive work (GCW), which is the work performed by the LV contributing to LV ejection during systole and is estimated by the myocardial work performed during shortening in systole, adding negative work during lengthening in isovolumetric relaxation (IVR); global wasted work (GWW), which is the worked performed by the LV that does not contribute to LV ejection and is estimated by the negative myocardial work performed during LV lengthening in systole, adding work performed during shortening in IVR; and global work efficiency (GWE), which is the constructive work divided by the sum of constructive and wasted work (GWE = GCW/(GCW + GWW), expressed in % between 0 and 100%).

All images were acquired and analysed by a single, experienced operator blinded to clinical data. To assess repeatability, intra-observer variability was evaluated in a random anonymized subset of 20 studies, re-analysed ≥2 weeks apart by the same operator blinded to the initial measurements (except for BP), yielding intraclass correlation coefficients (ICC) of 0.97 for GLS, 0.93 for GWE, 0.96 for GWW, 0.92 for GWI, and 0.96 for GCW. Test–retest reproducibility could not be assessed in this cohort and is acknowledged as a limitation.

### 2.3. Statistical Analysis

Statistical analysis was performed using SPSS v29.0 for Windows (SPSS Inc., 2005, Chicago, IL, USA). All continuous variables were tested for normality using histograms, Q–Q plots, and the Shapiro–Wilk test. Data for continuous variables are presented as mean (± standard deviation) or median (25–75% interquartile range). Categorical variables are presented as counts and percentages.

### 2.4. Groups Comparison and Correlation Analysis

Between-group differences were compared using the unpaired *t*-test, Mann–Whitney U test, and the Chi-square test as appropriate. Two groups were established for the purposes of analysis—one comprising participants who were genotype-positive (G+), and one other comprising participants who were genotype-negative (G−). We examined differences in myocardial work parameters between genotype groups. Pearson’s or Spearman’s rank tests were used for correlation analysis as appropriate.

### 2.5. Regression Analysis

Binary logistic regression was used to assess the value of echo parameters for the identification of pathogenic gene carriers. Univariate logistic regression analysis was used to find echocardiographic predictors of subclinical LV dysfunction. Multivariable logistic regression was performed to compare myocardial work parameters between groups. Variables significantly associated with gene carrier status in the univariate analysis were included in the multivariable logistic regression analysis. Regression diagnostics were performed to ensure the assumptions for logistic regression were satisfied.

### 2.6. ROC Curve Analysis

Sensitivity and specificity (calculated from the true/false, positive/negative classifications using standard definitions) were analysed using receiver-operator characteristic (ROC) curves in order to obtain the area under the curve (AUC) for different models utilizing parameters from univariate analysis that correlated with gene carrier status.

### 2.7. Statistical Significance

A two-tailed *p*-value of <0.05 was considered statistically significant.

## 3. Results

A total of 85 individuals were screened between 2018 and 2019. Out of those, 77 individuals met the relevant prespecified criteria. Baseline characteristics are presented in Table 1. There was no difference in age or history of hypertension, diabetes, or medical treatment between the two groups. The mean age was 36 ± 14 years, and 38 (49%) were female. Genotype-positive individuals carried LP/P variants in eight different genes (Table 2). No significant difference was found between G+ and G− individuals in mean systolic and diastolic BP, LVEF, GLS, GWI, or GCW.

### 3.1. Myocardial Work in Genotype-Positive, Phenotype-Negative Individuals

**Figure 2:**
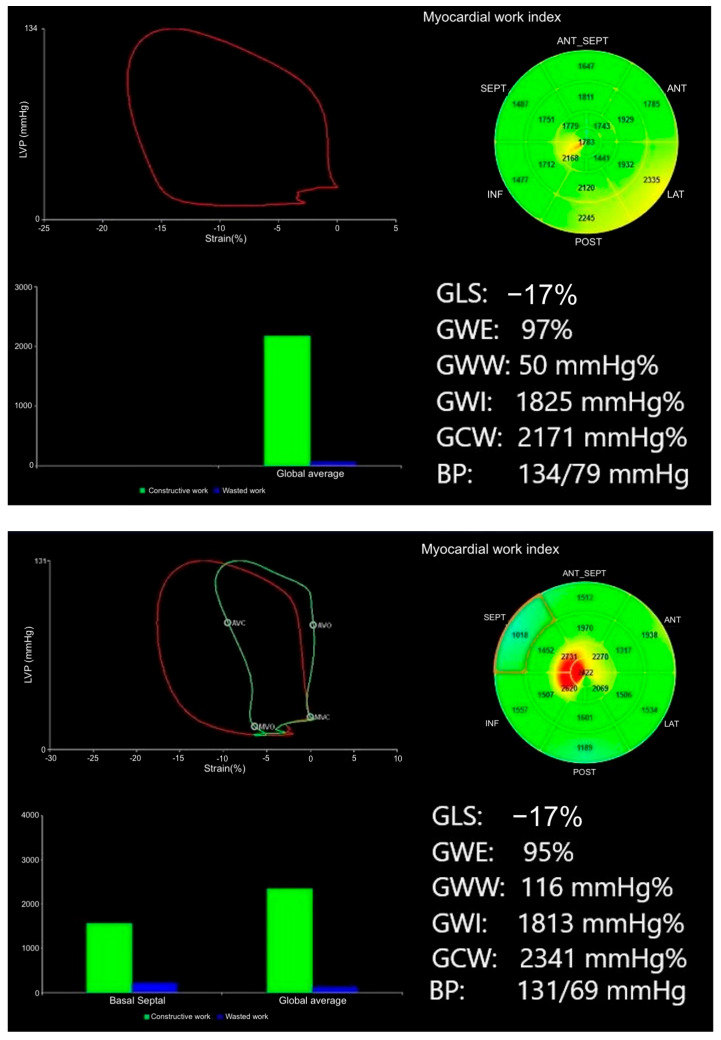
Representative images of MW analysis in G− (**top image**) vs. G+ individual (**bottom image**); despite similar GLS values, G+ individuals had lower GWE and higher GWW. In the pressure–strain loops, the red lines represent the global LV pressure–strain loop, while the green line (visible only in the bottom panel) represents a segmental loop (e.g., basal septal) contracting inefficiently. The bull’s-eye plots illustrate myocardial work index (GWI) distribution, and the bar graphs display constructive (GCW) vs wasted work (GWW).

There was a significant reduction in the GWE, with a GWE of 94% in G+ versus 96% in G− individuals (*p* < 0.001). Moreover, GWW was increased in the G+ with a GWW of 113 mmHg% vs. 80 mmHg% in G− individuals (*p* = 0.005) (Figure 2). Variables significantly associated with gene carrier status in the univariate analysis were included in the multivariable logistic regression analysis. The selected parameters are also recognized markers of LV longitudinal function and diastolic filling, supporting their biological plausibility. Global work efficiency (OR = 0.746, 95% CI: 0.560–0.994, *p* = 0.045) and GWW (OR = 1.012, 95% CI: 1.002–1.024, *p* = 0.047) remained significant predictors of gene carrier status after adjustment for traditional echocardiographic parameters (s′ septal, s′ average, e′ lateral, E max, and E/A) with known predictive significance in univariate analysis (Table 3). No variable included in the multivariable model had a significant bivariate correlation of ≥0.7 with *p* < 0.05 (Table 4).

### 3.2. Added Value of Myocardial Work Beyond Traditional Echocardiographic Parameters

**Figure 3:**
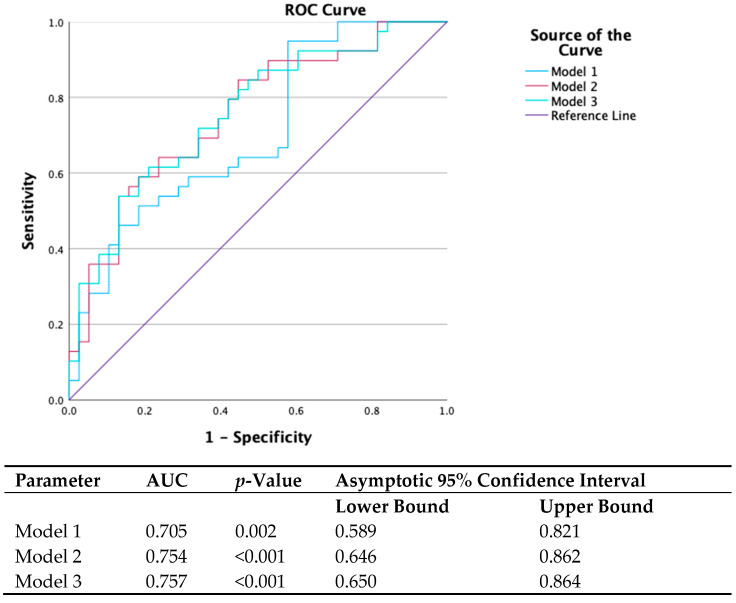
ROC curve analysis.

Receiver-operator characteristic curves were constructed for different models utilizing parameters from univariate analysis that correlated with gene carrier status (Figure 3). The following models were examined in ROC curve analysis:

- Model 1: s′ septal, s′ average, e′ lateral, E max, and E/A (parameters found to be correlated with gene status in univariate analysis).
- Model 2: Model 1 plus GWE.
- Model 3: Model 1 plus GWW.

Using ROC analysis, the AUC for differentiation between G+ and G− individuals of model 1 was 0.705 (95% CI: 0.59–0.82, *p* = 0.002), that of model 2 was 0.754 (95% CI 0.65–0.86, *p* < 0.001), and that of model 3 was 0.757 (95% CI 0.65–0.86, *p* < 0.001). In nested models’ comparison, the addition of GWE and GWW to the baseline model significantly increased the AUC of the model for identifying gene-positive individuals (*p* < 0.05 for both) (Figure 3).

### 3.3. Prognostic Value of Myocardial Work for the Development of Cardiomyopathy

During a median period of follow-up of 53 months (range of 31.50–69.00), 16 (21%) individuals expressed a cardiomyopathy phenotype, defined as reduced LV EF of <53%, or increased LV dimensions on follow-up echo (as defined in the inclusion criteria) (Table 5), with the vast majority belonging to the G+ group. Specifically, 1 G− and 15 G+ individuals (total N = 16) developed a phenotype [Phenotype Positive (+)], vs. 37 G− and 24 G+ individuals (total N = 61) who did not develop a phenotype [Phenotype Negative (−)] during follow-up. There was a significant correlation between increased baseline GWW, reduced GWE, and the expression of cardiomyopathic phenotype.

## 4. Discussion

Genetic testing of DCM cases may identify genotype-positive/phenotype-negative family members. However, gene status information may not always be available, and physicians may need to rely on clinical and echocardiographic markers of early disease in asymptomatic relatives; when genetic information is not available, the current European Society of Cardiology position statement and the American Heart Association guidelines advise periodic screening of first-degree relatives of DCM patients at variable intervals, but the level of evidence of the exact screening frequency and follow-up periods is low [1,6]. Importantly, a significant number of patients in early stages present with a non-dilated, hypokinetic stage, especially carriers of variants in genes known to be associated with non-dilated LV cardiomyopathy [7]. At this stage, institution of medical therapy can be considered, although the exact timing of this is not known [8].

It is possible that early disease can manifest in the absence of LV dilatation or overt dysfunction by conventional markers, and this underlines the need for sensitive markers of disease expression in DCM. Studies have shown that among asymptomatic relatives of patients with idiopathic or familial DCM screened by echocardiography, isolated LV enlargement without systolic dysfunction is an important precursor of inherited DCM [9]. Other studies have suggested that genotype-positive phenotype-negative relatives of DCM patients may have subtle systolic and diastolic dysfunction compared to healthy subjects, measured using LV GLS and peak left atrial strain, despite the presence of otherwise normal conventional echocardiographic parameters [10]. Moreover, although differences in tissue Doppler and strain parameters can be detected via echocardiography in first-degree relatives of patients with sarcomere gene variants, systolic dysfunction in subclinical DCM is generally very mild, with substantial overlap between strain measurements in carriers of genetic variants and normal controls [11]. Prognostic data on GLS in DCM patients are mainly available from CMR-derived feature tracking, and appear to be an independent predictor of mortality [12,13]. Data on echocardiography-derived GLS prognostic value in DCM are scarce. One study showed that GLS was a predictor of ventricular arrhythmias [14]. Another study showed that LV GLS may identify carriers of genetic variants for DCM with a normal LVEF at an early, subclinical stage [15]. A large study investigating the use of GLS to detect early DCM found that abnormal GLS was more common in DCM relatives compared to control subjects and was a predictor of LV EF deterioration, cardiac hospitalization, and death. However, abnormal GLS was mainly influenced by classic cardiovascular risk factors and family history of DCM instead of a proven genetic variant [16].

Compared to the aforementioned methods of early disease assessment, myocardial work has the potential to offer incremental value to myocardial function assessment by taking into account deformation as well as afterload through the interpretation of strain in relation to dynamic non-invasive LV pressure, reducing the potential influence of loading conditions, which happens with strain analysis [17,18]. Myocardial work and its prognostic significance have been extensively studied across various clinical conditions, such as hypertrophic cardiomyopathy, ST-elevation myocardial infarction, heart failure, valvular heart disease, and resynchronization therapy, with its potential applications continuing to expand [19,20,21,22,23,24]. In DCM patients, GWI has offered a better understanding of the relationship between LV remodelling and increased wall stress under different loading conditions [25]. A prospective study evaluated MW in patients with DCM compared to healthy controls and assessed MW before and after therapy. Patients with DCM showed significantly reduced MW compared to controls. Post-therapy, the GWI and 6 min walking distance improved, while LV EF and GLS did not change significantly, concluding that MW indices may be more sensitive in evaluating the therapeutic effects of medical therapy in DCM [26].

Our findings suggest that myocardial work analysis can uncover early energetic inefficiency and subtle dysfunction in DCM gene carriers, not apparent on conventional imaging. In our study, most echocardiographic parameters were within the normal range, and there were no significant differences between groups [27], whereas carriers of LP/P variants demonstrated significantly reduced myocardial work efficiency (GWE) and increased wasted work (GWW) compared with genotype-negative relatives, despite preserved EF and GLS. Importantly, GWE and GWW remained independent predictors of gene carrier status after adjustment for conventional echocardiographic markers, supporting the incremental value of myocardial work indices. In nested ROC models, adding GWE or GWW significantly improved discrimination beyond standard parameters, although overall AUCs remained modest (0.705–0.757), indicating that MW indices provide complementary rather than stand-alone information. In longitudinal follow-up, baseline reductions in GWE and elevations in GWW were associated with the subsequent development of a cardiomyopathy phenotype. This observation suggests that MW indices may have predictive value for early disease expression in genotype-positive individuals. It also illustrates the continuum in cardiomyopathy pathophysiology starting from an early subclinical phenotype, frequently undetected by conventional imaging methods, prior to progressing to overt disease [28,29]. These findings may also underscore the need to refine or expand existing normal ranges to account for genetic variability and subclinical myocardial changes. Incorporating genotype-specific data into future reference models may improve early detection and risk stratification in populations predisposed to cardiomyopathy [30,31].

While not intended as a replacement for genetic testing, MW indices can complement it by providing additional information about cardiac performance. This dual approach can enhance the overall diagnostic process for at-risk individuals. Validation in larger cohorts and longer-term follow-up with hard outcomes is required to evaluate whether these novel markers yield robust prognostic utility by identifying high-risk subgroups and whether they provide improved diagnostic accuracy in patient selection and timing of treatment initiation for familial DCM.

### Limitations

This was a single-centre, retrospective analysis of a modest cohort, limiting external validity—particularly for gene-specific subgroup analyses. Although group differences reached statistical significance, we recognize that considerable overlap exists across standard deviations, and, at this stage, should only be considered as adjunctive markers in clinical practice. Subgroup analysis for individual genes was not conducted due to the small study sample. We did not evaluate radial, circumferential, and rotational mechanics; atrial strain; or indices of diastolic function between groups. However, most participants had normal left atrial size and diastology. As the aim of this study was to predict gene carrier status among family members of affected individuals, comparisons were not made against phenotypically expressed DCM cases or normal controls. Although BP was standardized and contemporaneous with imaging, MW indices remain partly load-dependent. Medication effects (e.g., β-blockers and ACEi/ARB) were recorded but could not be fully disentangled. All imaging was performed by a single experienced operator, and we only report intra-observer repeatability. Test–retest reproducibility could not be assessed in this cohort. Although all participants were classified as asymptomatic at baseline according to standard clinical criteria, subtle or prodromal symptoms may not have been systematically recognized. The *p*-value for NSVT (0.087) between G+ and G− groups was borderline significant; it is not clear whether the NSVT cases reflect a subclinical phenotype in G+ individuals or simply fall within the background prevalence in the general population. Not all individuals underwent CMR scanning or follow-up echocardiography in our institution; therefore, no statistical analysis was performed between baseline and follow-up echocardiographic or CMR parameters. Finally, hard endpoints (HF hospitalization and mortality) were too infrequent for robust prognostic modelling. Larger, multicentre, longer studies are required.

## 5. Conclusions

Carriers of genetic variants for DCM with apparently normal LV function showed decreased myocardial work efficiency and increased wasted work compared to unaffected family members. Altered myocardial work indices were associated with the subsequent development of cardiomyopathy on follow-up, suggesting that GWW and GWE may be among the first markers to be affected in the natural history of DCM. Myocardial work analysis may provide incremental information for population-level risk stratification in at-risk relatives. Larger multicentre studies with longer follow-up are required before these indices can be considered for individual clinical decision making or early treatment initiation.

## Figures and Tables

**Table 1:** Baseline characteristics of the study population (G+ and G−).

Baseline Characteristics	G− (N = 38)	G+ (N = 39)	*p*-Value
Age (±SD)	33 (±12.8)	38 (±13.7)	0.082
Female (%)	18 (47%)	22 (55%)	0.503
BSA	1.91 (±0.2)	1.91 (±0.2)	0.963
Systolic BP (±SD)	121 (±15.8)	120 (±16)	0.656
Diastolic BP (±SD)	74 (±10.6)	72 (±11.3)	0.490
Smoking	2 (5.3%)	5 (12.5%)	0.249
Known CAD	0	0	-
Hyperlipidemia	1 (2.6%)	4 (10%)	0.175
Hypertension (%)	3 (7.9%)	4 (10)	0.747
Diabetes (%)	0	2 (5%)	0.165
ACEi/ARB (%)	4 (10.5%)	8 (20.5%)	0.230
BB	2 (5.3%)	4 (10%)	0.414
Loop diuretics	0	0	-
NSVT (%)	0	3 (7.5%)	0.087
AF	0	1 (2.5%)	0.320
QRS (ms)	97 ms	95 ms	0.459
**Myocardial work parameters**			
GWE (%)	96 (±1.5)	94 (±2.4)	<0.001
GWI (mmHg%)	1920 (±292)	1839 (±424)	0.125
GCW (mmHg%)	2250 (±325)	2217 (±474)	0.282
GWW (mmHg%)	80 (±35)	113 (±53)	0.001
**Traditional echo parameters**			
LVDD (mm)	52 (±6)	53 (±5)	0.363
LVDDi (mm/m^2^)	27 (±2)	28 (±3)	0.262
LVDVi	63 (±13)	61 (±12)	0.213
LVEF (%)	59 (±4)	58 (±4)	0.093
GLS (%)	18.7 (±1.7)	18.0 (±2.7)	0.089
Mitral s′ sep (cm/s)	8.3 (±1.4)	7.8 (±1.1)	0.019
Mitral s′ lat (cm/s)	10.7 (±2.6)	9.9(±2.5)	0.066
Mitral s′ avg	9.6 (±1.7)	8.8 (±1.5)	0.023
E max	73 (±15.4)	67 (±14.9)	0.061
A max	51(±16)	56(±16)	0.098
E/A ratio	1.6 (±0.5)	1.3 (±0.3)	0.005
E/e′	7.4 (±6.7)	6.2 (±1.9)	0.258
Mitral e′ septal	10.5 (±2.5)	9.6 (±2.3)	0.062
Mitral e′ lateral (cm/s)	15.2 (±3.9)	13.1 (±3.9)	0.011
AVC (ms)	405 (±44)	408 (±41)	0.735
LA area	18.6 (±3.7)	19 (±3.9)	0.626
LV posterior wall	7 (±1.2)	7 (±1.1)	0.722
IVS	8 (±1.4)	8 (±1.4)	0.647

BSA = body surface area; BB = beta-blockers; CAD = coronary artery disease; ACEi = angiotensin-converting enzyme inhibitor; ARB = angiotensin receptor blocker; NSVT = non-sustained ventricular tachycardia; AF = atrial fibrillation; GWE = global work efficiency; GWI = global work index; GCW = global constructive work; GWW = global wasted work; LVDD = left ventricular end-diastolic diameter; LVDDi = left ventricular end-diastolic diameter indexed to BSA; LVDVi = left ventricular end-diastolic volume indexed to BSA; LVEF = left ventricular ejection fraction; GLS = global longitudinal strain; s′ = systolic tissue Doppler velocity; E = peak early mitral inflow velocity; A = peak late mitral inflow velocity; e′ = early diastolic tissue Doppler velocity; AVC = aortic valve closure; LA = left atrial; IVS = interventricular septum.

**Table 2:** Genetic variants in G+ and G− individuals.

Gene	G+ (N = 39)
BAG3	3
DSP	17
FLNC	3
LMNA	3
RBM20	3
TNNT2	1
TPM1	1
TTN	8

**Table 3:** Regression analysis.

	Genotype Classification G+/G−			
Variable	OR (95% CI)	*p*-Value	OR (95% CI)	*p*-Value
	Unadjusted		Adjusted	
GWE	0.671 (0.511–0.881)	0.004	0.746 (0.560–0.994)	0.045
GWW	1.017 (1.005–1.028)	0.004	1.012 (1.002–1.024)	0.047

Binary logistic regression for GWE and GWW without and with adjustment. Adjusted model: adjusted for e′ lateral, s′ septal, s′ average, E/A, and E max; GWE: global work efficiency; GWW: global weighted work; OR: odds ratio.

**Table 4:** Correlates of myocardial work and other echo parameters.

	GWE	*p*-Value	GWI	*p*-Value	GCW	*p*-Value	GWW	*p*-Value
LVDD	−0.023	0.844	−0.209	0.069	−0.227	0.048	−0.050	0.663
LVDDi	−0.980	0.399	−0.115	0.320	−0.119	0.303	0.034	0.766
EF	0.653	<0.001	0.342	0.002	0.307	0.007	−0.453	<0.001
GLS	0.354	0.002	0.339	0.003	0.349	0.002	−0.275	0.015
S′ septal	0.428	<0.001	0.225	0.049	0.171	0.137	−0.373	<0.001
E max	0.072	0.532	0.267	0.019	0.239	0.036	−0.010	0.932
E/A ratio	0.259	0.023	0.093	0.419	0.078	0.498	−0.245	0.032
e′ lateral	0.212	0.064	0.040	0.731	0.020	0.865	−0.198	0.084
AVC	−0.074	0.524	−0.116	0.315	−0.045	0.700	0.105	0.365
DT	−0.084	0.467	−0.115	0.321	−0.088	0.446	0.021	0.857
LA area	0.099	0.394	−0.162	0.160	−0.155	0.180	−0.118	0.308
LVPWt	0.024	0.834	−0.187	0.104	−0.156	0.177	−0.069	0.553

Correlation coefficients for myocardial work parameters and other echo parameters. No variable included in the multivariable model had a significant bivariate correlation of ≥0.7 with *p* < 0.05. LVDD = left ventricular diastolic diameter; LVDDi = left ventricular diastolic diameter indexed; AVC = aortic valve closure; DT = deceleration time; LA = left atrium; LVPWt = left ventricular posterior wall thickness.

**Table 5:** Follow-up outcomes and baseline myocardial work (MW) parameters. MW parameters were measured at baseline prior to phenotype expression. “Cardiomyopathy phenotype” was defined a priori as LV EF < 53% and/or increased LV dimensions on follow-up echo (as defined in the inclusion criteria). Counts reflect the full cohort (N = 77) with follow-up data (G−, N = 38; G+, N = 39).

Genotype Group and Development of Cardiomyopathy Phenotype			
Follow-up data (N = 77)	G− (N = 38)	G+ (N = 39)	*p*-Value
Cardiomyopathy Phenotype Positive (+) (N = 16) *	1 (3%)	15 (38%)	<0.001
Cardiomyopathy Phenotype Negative (−) (N = 61)	37 (97%)	24 (62%)	<0.001
Myocardial work parameters and development of cardiomyopathy phenotype *			
**Myocardial work parameters**	**Phenotype Negative (−)** **(N = 61)**	**Phenotype Positive (+)** **(N = 16)**	***p*-Value**
GWE (%)	96 (±1.7)	94 (±3.0)	<0.001
GWI (mmHg%)	1915 (±337)	1706 (±417)	0.268
GCW (mmHg%)	2247 (±366)	2130 (±510)	0.091
GWW (mmHg%)	91 (±41)	121 (±65)	0.004

* Reduced LV EF < 53%, or increased LV dimensions on follow-up echo, as defined in the inclusion criteria.

## Data Availability

Original data are available upon request.

## Abbreviations

The following abbreviations are used in this manuscript:

A	Peak late mitral inflow velocity.
ACEi	Angiotensin-converting enzyme inhibitor.
AF	Atrial fibrillation.
ARB	Angiotensin receptor blocker.
AUC	Area under the curve.
AVC	Aortic valve closure.
BP	Blood pressure.
BSA	Body surface area.
CAD	Coronary artery disease.
CMR	Cardiac magnetic resonance imaging (cardiac MRI).
DCM	Dilated cardiomyopathy.
DT	Deceleration time.
E	Peak early mitral inflow velocity.
EDD	End-diastolic dimension.
EDVi	End-diastolic volume indexed.
EF	Ejection fraction.
e′	Early diastolic tissue Doppler velocity.
G+	Genotype positive.
G−	Genotype negative.
GCW	Global constructive work.
GLS	Global longitudinal strain.
GWE	Global work efficiency.
GWI	Global work index.
GWW	Global wasted work.
IVS	Interventricular septum.
LA	Left atrium.
LP/P	Likely pathogenic/pathogenic.
LV	Left ventricle/ventricular.
LVDD	Left ventricular end-diastolic diameter.
LVDDi	Left ventricular end-diastolic diameter indexed to BSA.
LVDVi	Left ventricular end-diastolic volume indexed to BSA.
LVEF	Left ventricular ejection fraction.
LVPWt	Left ventricular posterior wall thickness.
MW	Myocardial work.
NSVT	Non-sustained ventricular tachycardia.
OR	Odds ratio.
ROC	Receiver-operator characteristic.
s′	Systolic tissue Doppler velocity.

## Appendix A

Cardiomyopathy gene panel (120 genes): A2ML1, ABCC9, ACADVL, ACTC1, ACTN2, AGL, ANKRD1, ATP5E, BAG3, BRAF, CALR3, CASQ2, CAV3, CBL, COA5, CPT2, CRYAB, CSRP3, CTF1, CTNNA3, DES, DMD, DMPK, DNAJC19, DNM1L, DOLK, DSC2, DSG2, DSP, DTNA, EMD, EYA4, FHL1, FHL2, FKTN, FLNC, FOXRED1, GAA, GATAD1, GBE1, GLA, GLB1, GUSB, HFE, HRAS, ILK, JPH2, JUP, KRAS, LAMA4, LAMP2, LDB3, LMNA, MAP2K1, MAP2K2, MIB1, MMACHC, MRPL3, MUT, MYBPC3, MYH11, MYH6, MYH7, MYL2, MYL3, MYLK2, MYOM1, MYOZ2, MYPN, NEBL, NEXN, NF1, NRAS, PCCA, PCCB, PDLIM3, PIK3CA, PIK3R2, PKP2, PLN, PNPLA2, PRDM16, PRKAG2, PTPN11, RAF1, RASA1, RBM20, RIT1, RYR2, SCN5A, SCO2, SGCD, SHOC2, SLC22A5, SLC25A20, SLC25A3, SLC25A4, SOS1, SPRED1, STAMBP, STK4, SYNE1, SYNE2, TAZ, TCAP, TGFB3, TMEM43, TMEM70, TMPO, TNNC1, TNNI3, TNNT2, TPM1, TRIM63, TSFM, TTN, TTR, TXNRD2, VCL, XK.
